# A Wafer-Level Fabricated Heating–Vacuum Micro-Platform with Resonant MEMS Monolithically Integrated

**DOI:** 10.3390/mi16020214

**Published:** 2025-02-13

**Authors:** Kaixuan He, Rui Feng, Yu Zheng, Lijian Guo, Qichao Liao, Dongfang Song, Yuan Xiang, Xinxin Li

**Affiliations:** 1 State Key Laboratory of Integrated Chips and Systems, School of Microelectronics, Fudan University, Shanghai 200433, China; 17112020020@fudan.edu.cn; 2 East China Institute of Photo-Electron IC, Bengbu 233000, China; richardfeng85@gmail.com (R.F.); 13865015762@163.com (Y.Z.); spring_dong@163.com (L.G.); anyuqier@163.com (Q.L.); songdf1988@163.com (D.S.); 18715520925@163.com (Y.X.); 3 State Key Laboratory of Transducer Technology, Shanghai Institute of Microsystem and Information Technology, Chinese Academy of Sciences, Shanghai 200050, China

**Keywords:** resonant MEMS, vacuum, wafer-level integrated, micro-oven control

## Abstract

This paper presents a silicon-based wafer-level vacuum packaging platform with a monolithically integrated micro-oven. This system provides vacuum and constant temperature operating conditions to improve the performance of resonant micro-electro-mechanical systems (MEMS) devices. Based on a three-layer wafer-level vacuum packaging process, the platform integrates a silicon thermistor, a thermal isolation structure, and a heater with the addition of a mask and an additional silicon wafer. This wafer-level vacuum-packaging platform achieved a vacuum level of approximately 6 mTorr. Due to the micro-oven, the temperature coefficient of the resonant frequency for the MEMS resonator was reduced by 48 times, and the temperature coefficient of the quality factor was reduced 19 times within the temperature range of −40 °C to 80 °C. The heater of the micro-oven consumed about 364 mW of power when the ambient temperature was −40 °C and the temperature controlled by the micro-oven was 100 °C. This method enables the wafer-level integration of the thermistor, thermal isolation structure, heater, and vacuum-packaged resonator, offering advantages such as low cost, efficient batch production, and high performance.

## 1. Introduction

Micro-Electro-Mechanical Systems (MEMS) utilize semiconductor manufacturing technology to achieve the miniaturization and integration of mechanical structures, offering advantages such as small size, low power consumption, and mass production [1,2]. However, movable structures in MEMS are extremely small and highly susceptible to external environmental influences. Factors such as temperature, vibration, and air damping can cause performance degradation or failure of MEMS devices [3,4,5,6,7,8,9]. Minimizing the impact of external environmental factors on MEMS devices remains a key challenge. Resonant MEMS devices, such as MEMS gyroscopes and MEMS oscillators, often require operation in a vacuum environment to reduce air-damping effects. This improves the quality factor (Q-factor) and enhances the signal-to-noise ratio (SNR) of the resonators [10,11,12,13,14,15]. To meet the stringent vacuum requirements of resonant MEMS devices, wafer-level vacuum packaging technology has seen significant advancements, achieving vacuum levels as low as 10 mTorr to 100 mTorr, which has significantly contributed to enhancing the performance of resonant MEMS devices [11,12,13,16,17]. However, addressing air damping alone is insufficient, as temperature fluctuations also critically impact device performance. The temperature fluctuations can cause variations in the Young’s modulus of silicon. These changes directly impact critical parameters, such as the resonant frequency and Q-factor, leading to significant performance degradation. Therefore, to further enhance the performance and stability of resonant MEMS devices, it is necessary to address the influence of temperature on device performance in addition to wafer-level vacuum packaging technology.

Micro-oven control, as an active temperature-compensation method, stabilizes the device at a constant temperature that is higher than the maximum operating ambient temperature by heating. It is a key technological approach to solving the impact of temperature fluctuations on the performance of resonant MEMS devices [18]. The implementation of micro-oven control mainly includes the following approaches. The first approach is to monolithically integrate MEMS structures with heaters at the wafer-level, thereby enabling constant temperature control of the MEMS structures [19,20,21]. The second approach is to fabricate a substrate wafer with an integrated thermistor, thermal isolation structure, and heater. The MEMS structure dies are then bonded to the substrate wafer, and wafer-level vacuum packaging is achieved through wafer-level bonding [3,22]. The third approach is to bond the MEMS chip to a packaging substrate that is equipped with a thermistor, a thermal isolation structure, and a heater [23,24], with micro-oven control implemented at the package level. Among these methods, the first method heats the MEMS structures and maintains them at a constant temperature, thereby achieving low power consumption. However, during operation, the chip substrate temperature still fluctuates with ambient temperature variations, leading to thermal stress transmission to the chip, which may degrade device performance. The second method is considered a quasi-wafer-level integration approach, which, while achieving wafer-level vacuum packaging, also has the functions of micro-oven control and stress isolation. However, bonding the MEMS structure dies to the substrate wafer still requires individual die operations, and the complexity of the manufacturing process, low batch production, and high cost are disadvantages. The third type is a package-level integration method, which is relatively simple but lacks the advantages of large-scale wafer-level processes and low cost.

This paper proposes a silicon-based wafer-level vacuum packaging platform with an integrated on-chip micro-oven. Based on a three-layer wafer-level vacuum packaging process, this approach just incorporates an additional mask and a wafer. Using deep silicon etching and fusion bonding for embedded structures and a clever grinding release process, it enables the wafer-level integration of a thermistor, a thermal isolation structure, a heater, and a MEMS resonator. This method uniquely combines wafer-level vacuum packaging with a monolithically integrated micro-oven, addressing both air damping and temperature fluctuations in a single process. Unlike existing methods, it enables high-performance MEMS devices with wafer-level fabrication, reducing manufacturing complexity and cost. Moreover, this method enables uniform heating of the entire chip structure and, in conjunction with thermal isolation structures, effectively isolates packaging stress. This will further enhance the overall performance of the MEMS device.

## 2. Design

### 2.1. Platform Structure

**Figure 1:**
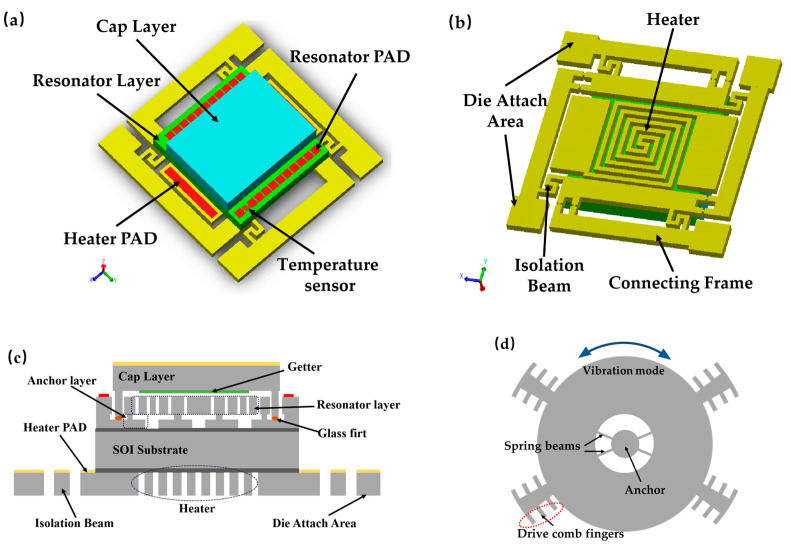
Schematic diagram of the wafer-level vacuum-packaged platform with an integrated on-chip micro-oven. (**a**) Front; (**b**) back; (**c**) cross-section; (**d**) Schematic diagram of resonant structure.

The schematic diagram of the wafer-level vacuum-packaged platform with an integrated on-chip micro-oven is shown in Figure 1. This structure primarily consists of a cap layer (silicon wafer 1), a MEMS resonator layer (SOI wafer 1 and SOI wafer 2), and a heating and spring isolation layer (silicon wafer 2). A cavity is fabricated into the cap wafer to provide space for the movement of the resonant structures. Additionally, a getter thin film is deposited inside the cavity to enhance the vacuum level of the subsequent packaging process. The cap wafer is bonded to the MEMS resonator wafer using glass frit bonding. The MEMS resonator is composed of two SOI wafers. SOI wafer 1 is used to form the anchors and electrical interconnections, while SOI wafer 2 is used to form the movable structures. The resonant structure is a type of vibrating wheel structure, as shown in Figure 1d. The vibrating wheel structure comprises key components such as anchor, spring beams, and driving comb fingers. Subsequent research focused on the in-plane vibration mode. A silicon thermistor is also fabricated on the movable structure layer. The MEMS resonator is bonded to the heating and spring isolation layer through silicon fusion bonding. The heating and isolation layer contains the silicon heater, thermal and stress isolation spring structure, and die attachment structures for packaging. To reduce parasitic resistance, the MEMS device layers use low-resistivity silicon with a resistivity of 0.01 Ω·cm to 0.02 Ω·cm. The thickness of the SOI substrate is minimized to 380 μm to reduce the thermal capacity and lower the power consumption of the micro-oven. Silicon wafer 2 is used as the heater, with its resistivity ranging from 0.005 Ω·cm to 0.025 Ω·cm, and the resistance of the heater within a range of several tens of ohms. The main parameters of the platform structure are shown in Table 1. The detailed fabrication process is described in Section 3.

### 2.2. Theoretical Analysis and Thermal Optimization

Power consumption is a key index of the micro-oven. To reduce its consumption of heating power, it is essential to minimize the MEMS die’s thermal losses, which primarily originate from three sources: (1) thermal conduction losses through the adhesive layer and gold interconnect wires between the die and the package; (2) thermal radiation losses from the die surface to the surrounding environment; (3) thermal convection losses from the die surface to the surrounding air. A one-dimensional lumped-parameter thermal model was employed to analyze the power consumption [25].

The conductive thermal resistance (*R_th_*) can be expressed using the following equation:

(1) $$R_{th}=\frac{l}{kA_{c}},$$

where *k* represents the thermal conductivity, *l* is the distance over which heat is conducted, and *A*_c_ is the cross-sectional area over which heat is conducted.

The expression for radiative resistance (*R_rad_*) is given as follows:

(2) $$R_{rad}=\frac{1}{h_{rad}A_{s}},$$

where *h_rad_* = *εσ*(*T_s_* + *T_surr_*)(*T_s_*^2^ + *T_surr_*^2^) represents the radiation heat transfer coefficient, *A_s_* is the radiative surface area, *T_s_* is the temperature of the radiating surface, and *T_surr_* is the temperature of the surrounding environment. *ε* is the emissivity, representing the radiative property of the surface, with a value range of 0 ≤ *ε* ≤ 1. *σ* is the Stefan–Boltzmann constant, *σ* = 5.67 × 10^−8^ W/(m^2^·K^4^).

The convective resistance (*R_conv_*) can be determined using the following equation:

(3) $$R_{conv}=\frac{1}{h_{conv}A_{s}},$$

where *h_conv_* = *q*”/(*T_s_* − *T_∞_*) is the convection heat transfer coefficient, *A_s_* is the surface area, *q*” is the convective heat flux density, *T_s_* is the temperature of the die surface, and *T_∞_* is the temperature of the air fluid. For natural convection, the typical convection heat transfer coefficient ranges from 2 W/(m^2^·K) to 25 W/(m^2^·K) [26].

**Figure 2:**
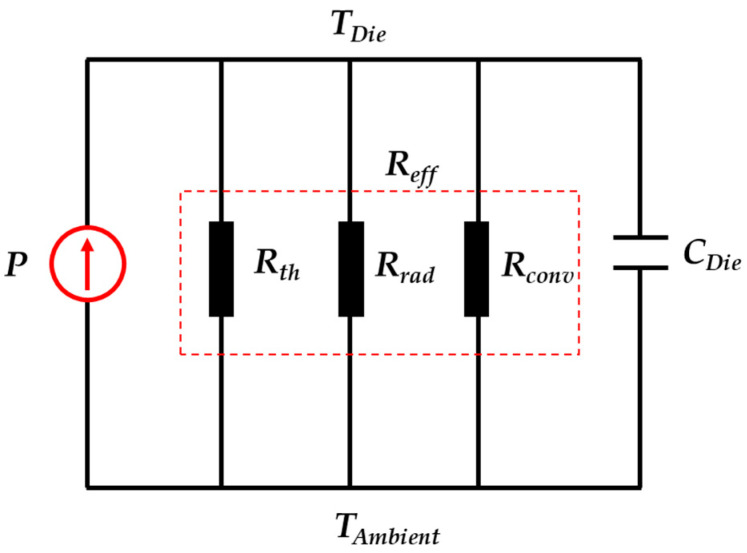
Thermal equivalent circuit of the micro-oven.

The thermal equivalent circuit of the micro-oven control system is shown in Figure 2. The ambient temperature around the die is *T_Ambient_*, and the target temperature of the micro-oven-controlled die is *T_Die_*. The heating power at a steady state is *P*. The heat capacity of the die is defined as *C_Die_*. The thermal resistances of the die for conduction, radiation, and convection are denoted as *R_th_*, *R_rad_*, and *R_conv_*, respectively. The effective thermal resistance is defined as *R_eff_*.

The power consumption at steady state can be expressed as follows:

(4) $$P=\frac{∆T}{R_{eff}}=\frac{T_{Die}-T_{Ambient}}{R_{eff}}.$$

Assuming that natural convection heat transfer coefficient of the micro-oven is set to the average value of 10 W/(m^2^·K), and the radiation emissivity *ε* is set to the maximum value of 1, when the ambient temperature around the micro-oven is −40 °C, and the micro-oven control target temperature is set to 100 °C, the following results were obtained using the previously mentioned formulas. The conductive thermal resistance, radiative thermal resistance, and convective thermal resistance were approximately 319 K/W, 4651 K/W, and 1849 K/W, respectively. The heating power was about 544 mW.

**Figure 3:**
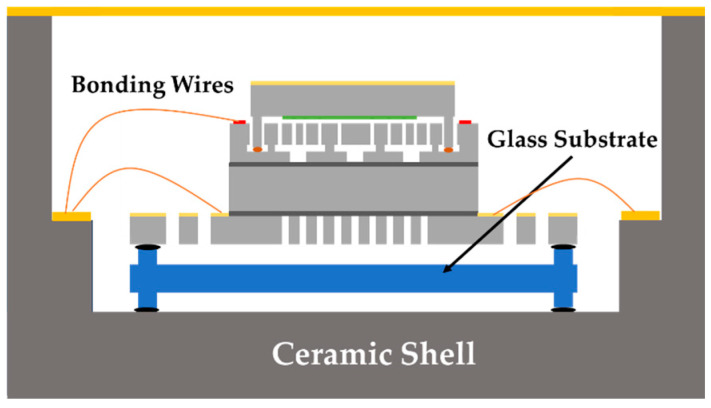
Schematic diagram of the micro-oven-controlled die packaging.

**Figure 4:**
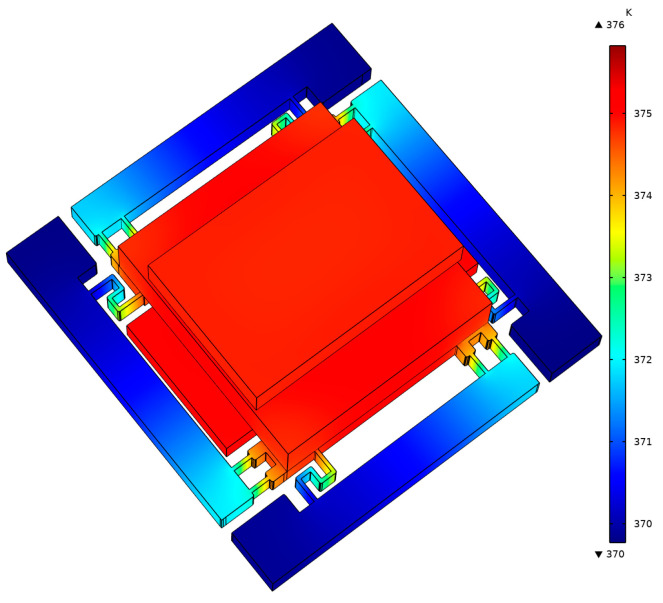
Simulation results of the temperature distribution of the micro-oven-controlled die.

The above calculations show that the primary source of thermal loss in the micro-oven comes from thermal conduction in the die attachment areas. To further reduce the power consumption of the micro-oven, a glass substrate with double-side symmetric columnar legs was added between the die and the package. The glass substrate was first attached to the shell, and then the micro-oven-controlled die was attached to the glass substrate, as shown in Figure 3. This method increased the conductive thermal resistance to 534 K/W, and the theoretical heating power at the extreme environmental temperature of −40 °C could be reduced to 368 mW. The simulation results using COMSOL 5.2 reveal that the temperature distribution of the resonator part was relatively uniform, as shown in Figure 4.

## 3. Fabrication

**Figure 5:**
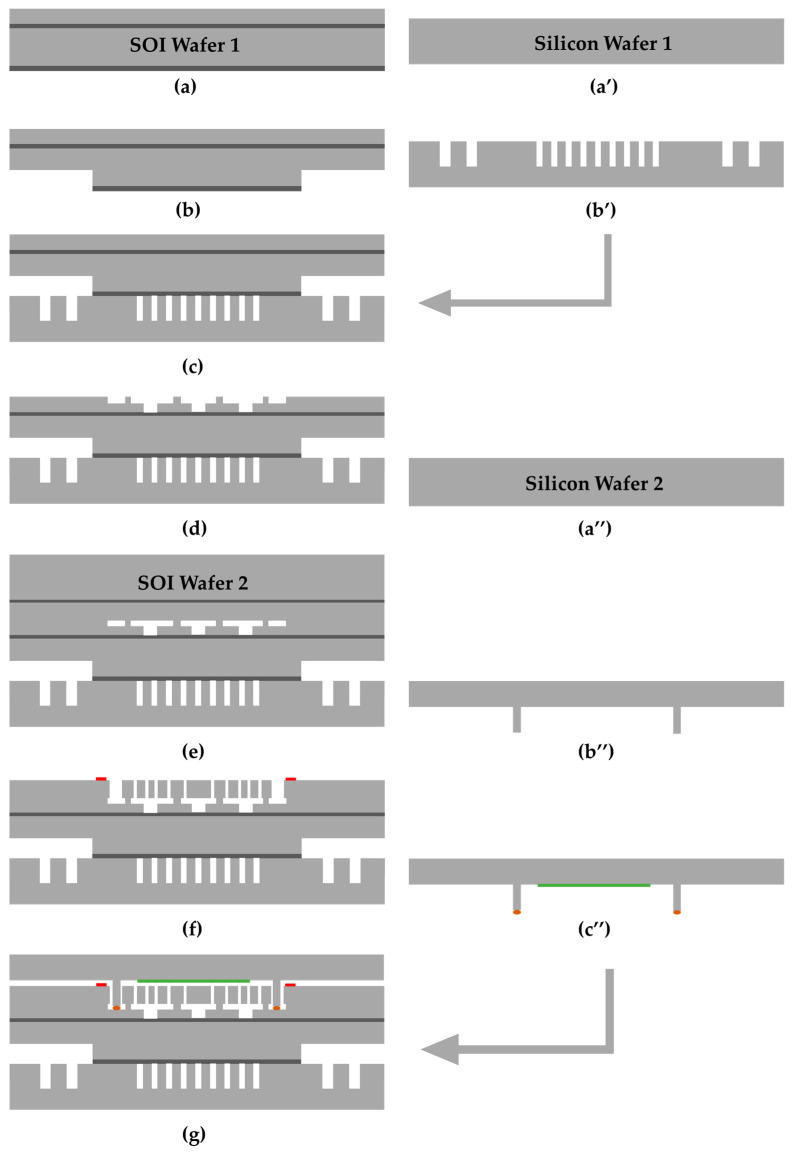

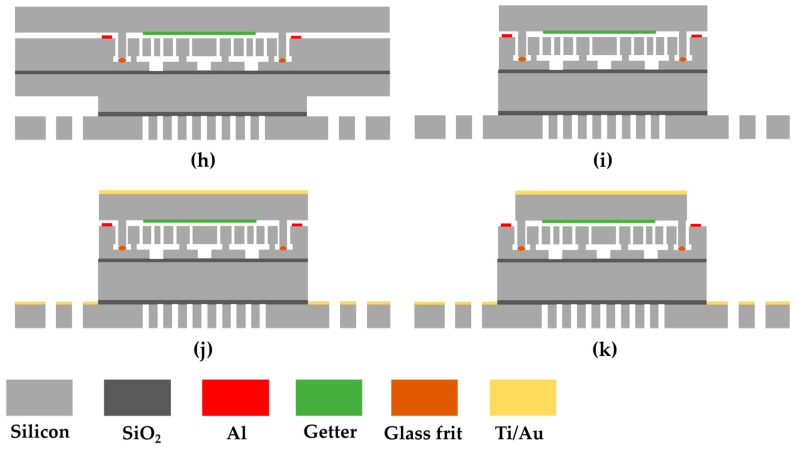
Process flow diagram for the fabrication of a silicon-based wafer-level vacuum packaging platform with an integrated on-chip micro-oven: (**a**) SOI wafer 1; (**b**) trench etching; (**a’**) silicon wafer 1; (**b’**) isolation and heating structure etching; (**c**) fusion bonding; (**d**) anchor and cavity etching; (**e**) fusion bonding; (**f**) MEMS structure etching and metal pads fabrication; (**a’’**) silicon wafer 2 used for cap; (**b’’**) KOH etching; (**c’’**) glass frit printing and getter deposition; (**g**) wafer-level vacuum bonding; (**h**) releasing isolation and heating structures using grinding; (**i**) suspended dicing; (**j**) metal deposition; (**k**) suspended dicing.

As described in Section 2.1, the platform structure consists of four wafers: SOI wafer 1, SOI wafer 2, silicon wafer 1, and silicon wafer 2, as shown in Figure 5. The main process is described as follows:

1. The process flow begins from SOI wafer 1 (Figure 5a). Deep trenches are etched on the back of the SOI wafer 1 substrate (Figure 5b) using deep reactive ion etching (DRIE), which will later expose the heater pads and die attachment structures through the dicing process. The depth of the trench is approximately 50 μm.
2. Meanwhile, isolation and heating structure patterns are fabricated on silicon wafer 1 through photolithography and the DRIE process (Figure 5a’,b’). The etching depth is about 250 μm.
3. SOI wafer 1 and silicon wafer 1 are bonded together using fusion bonding (Figure 5c). Fusion bonding annealing is performed at a temperature of 1100 °C for a duration of 4 h.
4. The device layer of SOI wafer 1 is processed to form anchors, cavities of the movable structures, and interconnects for the resonator (Figure 5d). The thickness of the SOI device-layer is about 10 μm. First, shallow cavities are etched using DRIE to form anchors, with an etching depth of approximately 5 μm. Subsequently, air isolation is achieved by performing DRIE between the silicon interconnects down to the buried oxide layer.
5. SOI wafer 2 is introduced, and subsequently, fusion bonding is performed with SOI wafer 1 (Figure 5e), wherein the device layer of SOI wafer 2 serves as the MEMS movable structure layer. The device layer of SOI wafer 2 is about 50 μm. The substrate of SOI wafer 2 is removed via grinding and KOH wet etching. Subsequently, MEMS movable structures are fabricated on the device layer using photolithography and DRIE processes, and metal pads are deposited via sputtering on the same layer (Figure 5f).
6. On silicon wafer 2 (Figure 5a’’), a KOH etching process is used to create the cap cavity structure and the bonding seal ring (Figure 5b’’). The depth of the cavity is approximately 100 μm. Glass frit is then printed onto the seal ring by using the screen-printing process, and a Ti-based getter film is deposited inside the cavity via electron beam evaporation (Figure 5c’’).
7. The cap wafer is then bonded with the MEMS movable structure layer using glass frit bonding in a vacuum bonder, thereby achieving wafer-level vacuum packaging (Figure 5g). The bonding temperature is maintained at 450 °C for 1 h to fully activate the getter, and the vacuum pressure in the bonding chamber is set to less than 0.075 mTorr.
8. The back of silicon wafer 1 is ground using an 8000-grit grinding wheel to release the isolation and heating structure (Figure 5h). The grinding equipment is equipped with an online thickness detection system, which ensures the grinding thickness accuracy is maintained within ±3 μm. The final remaining thickness of the silicon wafer 1 is approximately 180 μm.
9. Suspended dicing is performed to remove the silicon above the heater pads (Figure 5i), and then Ti/Au is deposited using sputtering to form the heater contact pads (Figure 5j). Finally, a second suspended dicing step is performed to expose the contact pads of the movable structures (Figure 5k).

**Figure 6:**
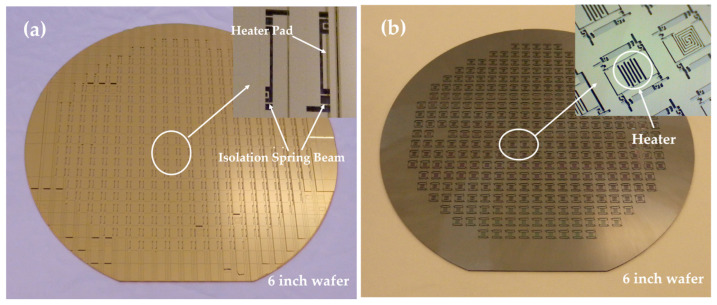
Photographs of the process. (**a**) Photograph of the front of the wafer after the first suspended dicing process; (**b**) photograph of the back of the wafer after the grinding process.

Figure 6 shows photographs taken during the wafer fabrication. Figure 6a is a photograph of the front of the wafer. After the first suspended dicing, the heater contact areas and isolation spring beams are exposed, and ohmic contact metal forms on the contact areas. Figure 6b shows a photograph of the back of the wafer. Through a meticulous grinding process, the embedded isolation and heating structures are released.

**Figure 7:**
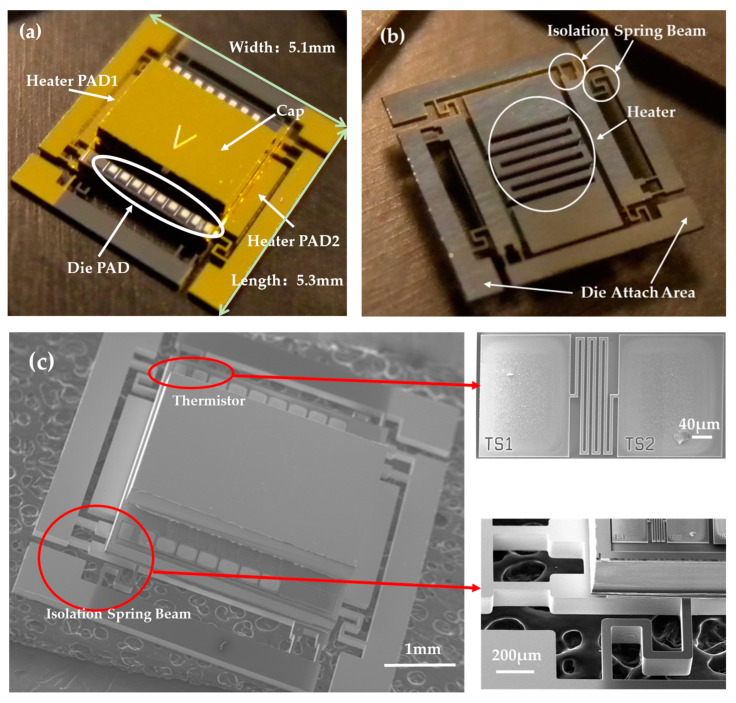
Photographs of the micro-oven-controlled die. (**a**) Photograph of the front of the die; (**b**) photograph of the back of the die; (**c**) detailed SEM photograph of the die.

Figure 7 shows photographs of the fabricated die. Figure 7a is a photograph of the front of the die, highlighting structures such as the cap, heater pads, and the die pad. Figure 7b is a photograph of the back of the die, displaying features including the isolation spring beams, heater, and die attachment areas for packaging. Figure 7c presents a scanning electron microscopy (SEM) image of the die, where the temperature sensor has a folded silicon thermistor located on the movable structure layer.

**Figure 8:**
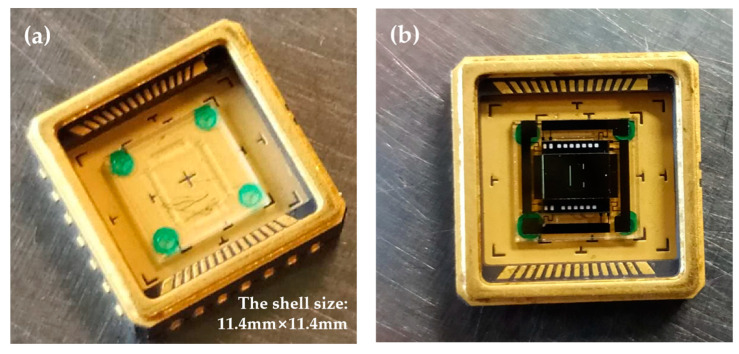
Photographs of the package. (**a**) Photograph after the glass substrate was attached to the package; (**b**) photograph after the die was attached to the glass substrate.

Figure 8 shows photographs of the packaging. To reduce thermal conduction losses, a BF33 glass with high thermal resistance was added between the die and the package. The glass substrate was adhered to the package using a four-point adhesive method, as shown in Figure 8a. The MEMS die was then attached to the glass substrate using the same four-point adhesive approach, as illustrated in Figure 8b. Finally, a metal cap was sealed using a laser welding process.

## 4. Results and Discussion

### 4.1. Wafer-Level Vacuum Packaging Characterization

#### 4.1.1. Package Pressure

The residual gas inside the cavity of the die was analyzed by SAES Group. The SAES’s residual gas analyses (RGA) equipment based on mass spectrometers are able to measure pressures down to 10^−4^ mbar in sealed cavities with internal volumes lower than 1 mm^3^ [27], and the results are shown in Table 2. The wafer-level vacuum packaging achieved a vacuum of approximately 6 mTorr, which is slightly better compared to previously reported results [12,16]. No reactive gases were detected in the residual gas composition, indicating that the getter was fully activated and effectively absorbed gases. The proportion of Ar was over 96%, which is partly due to the presence of Ar in the air and partly because Ar was used as a purging gas during the vacuum bonding process. Additionally, the getter did not adsorb inert gases like Ar. Other residual gases, including CH_4_, which accounted for 3.58%, are suspected to have been released from the glass frit bonding process.

#### 4.1.2. Q-Factor of the Resonator

**Figure 9:**
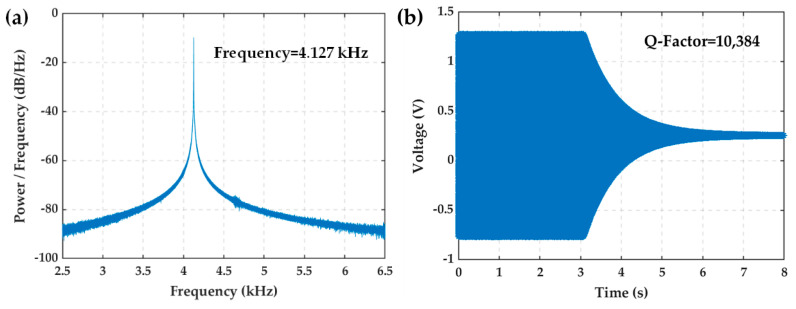
Resonant frequency and Q-factor results at room temperature. (**a**) Frequency sweeping curves; (**b**) ring-down curves.

A dynamic signal analyzer, along with external driving and readout circuits, was used to perform frequency sweeping at room temperature. The Q-factor of the MEMS resonator was obtained using the time decay method, as shown in Figure 9. The resonant frequency of was 4.127 kHz (Figure 9a), and the Q-factor was 10,384 (Figure 9b). After an 18-month period, the MEMS resonator was re-tested, yielding a Q-factor of 11,136, which exhibited no significant deviation from the initial measurement. This result substantiates the long-term stability of the wafer-level vacuum packaging.

### 4.2. Micro-Oven Characterization

#### 4.2.1. Thermistor Calibration

**Figure 10:**
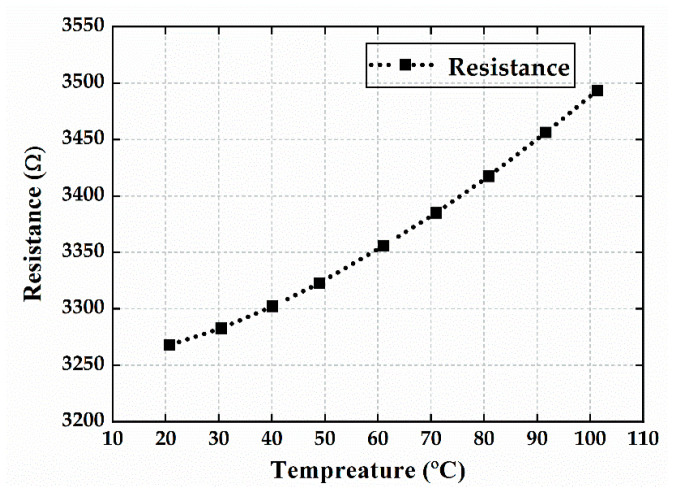
Resistance–temperature characteristics of the thermistor.

The on-chip silicon thermistor was calibrated using a temperature-controlled oven, and the resistance of the thermistor was measured at different temperatures using the four-point method. Figure 10 shows the resistance–temperature characteristics of the thermistor from 20 °C to 100 °C. As the temperature increased, the resistance also increased, though not in a completely linear trend. The average change rate was approximately 2.82 Ω/°C, resulting in a temperature coefficient of resistivity (TCR) of about 0.086%/°C, which is lower than the 1% TCR typically found in bulk monocrystalline silicon [28]. This is because the thermistor and the resonator were fabricated on the same silicon layer, and the chosen monocrystalline silicon was heavily doped with boron. This TCR is characteristic of boron-doped low-resistivity silicon.

#### 4.2.2. Temperature Coefficient of Resonant Frequency

**Figure 11:**
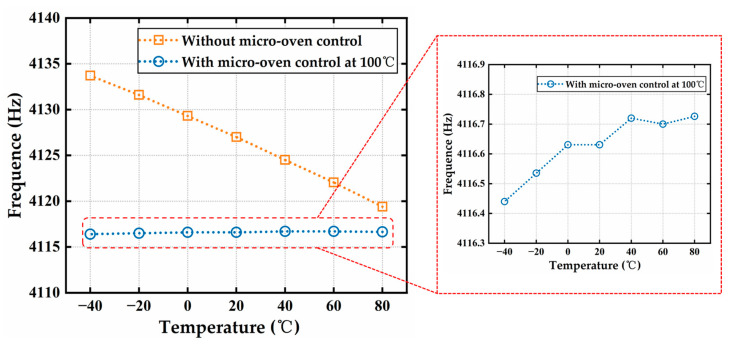
Curves of the resonant frequency under conditions with and without micro-oven control at a temperature range from −40 °C to 80 °C.

The MEMS resonator was tested for frequency variation in a temperature-controlled oven within the temperature range from −40 °C to 80 °C at 20 °C intervals. Each test point was stabilized for 1 h before the measurements. The test results are shown in Figure 11. Without micro-oven control, the resonator exhibited a negative temperature coefficient of the resonant frequency (TCF) of −28.9 ppm/°C, which is slightly lower than the TCF of −30 ppm/°C for conventional monocrystalline silicon [29,30]. This is attributed to the use of heavily boron-doped monocrystalline silicon in the resonator structure, which resulted in a reduced temperature coefficient [22,31]. As the temperature increased, the resonant frequency decreased, mainly due to the reduction in Young’s modulus of the monocrystalline silicon. This decrease in Young’s modulus caused the structure to soften, ultimately lowering its resonant frequency. After enabling micro-oven control, the MEMS resonator was heated to 100 °C, and the same testing method was applied. The TCF decreased to 0.6 ppm/°C, nearly 48 times lower than the result without micro-oven control. The previously reported TCF results ranged from 0.22 ppm/°C to 1 ppm/°C [19,21,22], which are similar to the result of this study.

#### 4.2.3. Temperature Coefficient of the Q-Factor

**Figure 12:**
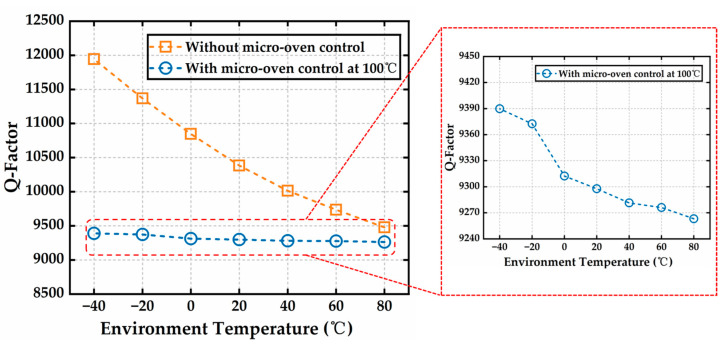
Curves of the Q-factor under the conditions with and without micro-oven control at a temperature range from −40 °C to 80 °C.

The Q-factor of the MEMS resonator was tested using the same method, and the results are shown in Figure 12. Without micro-oven control, the Q-factor decreased as the temperature increased, with an average temperature coefficient of the Q-factor (TCQ) of −1974.2 ppm/°C. This is because, as the temperature rose, the resonant frequency of the resonator decreased, and thermal thermoelastic damping increased, both of which contributed to the reduction in the Q-factor. Under micro-oven-controlled conditions, Q-factor tests were performed at different temperatures, and the TCQ was −107.7 ppm/°C, which is nearly 19 times lower than the result without micro-oven control. The remaining TCQ may be attributed to the effect of the temperature increase on gas damping. As the temperature rose, gas molecules adsorbed on the surface of the seal cavity were released, leading to an increase in gas damping.

#### 4.2.4. Power Consumption

**Figure 13:**
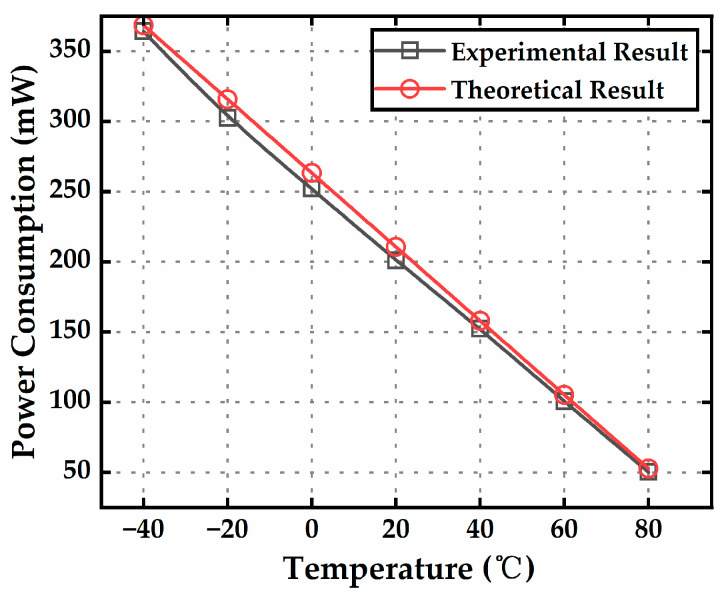
Theoretical and tested power consumption of the micro-oven at different ambient temperatures when the micro-oven was controlled at 100 °C.

The MEMS resonator was oven-controlled at 100 °C, and the power consumption of the heater was tested at different ambient temperatures, with a comparison made to theoretical results, as shown in Figure 13. The experimental results show high agreement with the theoretical calculations. When the ambient temperature was at −40 °C, the maximum power consumption of the oven-controlled MEMS reached 364 mW, which is very close to the theoretical value of 368 mW.

## 5. Conclusions

A novel wafer-level fabricated heating–vacuum micro-platform with a monolithically integrated resonant MEMS is demonstrated in this paper. Building upon the traditional three-layer wafer-bonding vacuum packaging process, the wafer-level integration of a thermistor, a heater, an isolation structure, and a MEMS resonator is achieved by adding only a mask and an additional silicon wafer, combined with deep silicon-etched embedded structures and a clever grinding release process. This platform achieved 6 mTorr high-vacuum wafer-level packaging by integrating a getter film. This packaging vacuum can meet the working requirements of most resonant MEMS devices. Within the temperature range of −40 °C to 80 °C, the TCF and TCQ of the MEMS resonator were reduced from −28.9 ppm/°C to 0.6 ppm/°C and from −1974.2 ppm/°C to −107.7 ppm/°C, respectively, which verifies the feasibility of the micro-oven control. In the future, better temperature performance can be obtained through high-precision closed-loop temperature control circuits. This platform leverages the advantages of wafer-level integration, making it suitable for mass production. In addition to providing wafer-level vacuum packaging and on-chip micro-oven, it also achieves stress isolation through isolation springs, which significantly enhances the performance of MEMS devices. The platform is applicable to a wide range of resonant MEMS devices, particularly MEMS inertial sensors such as gyroscopes and accelerometers. Wafer-level vacuum packaging effectively improves the signal-to-noise ratio, while micro-oven control mitigates the impact of environmental temperature fluctuations on device stability and performance. This addresses the challenge of environmental sensitivity in MEMS inertial devices and has the potential to achieve navigation-grade precision. Consequently, this platform will further expand the application fields for high-performance MEMS devices.

## Figures and Tables

**Table 1:** The main parameters of the platform structure.

Parameters	Values	Units
Thickness of cap wafer	525	μm
Depth of the cavity in the cap wafer	100	μm
Area of the cap	6.64 × 10^6^	μm^2^
Thickness of the resonator	50	μm
Thickness of the anchor layer	10	μm
Thickness of the SOI substrate	380	μm
Area of the SOI substrate	1.05 × 10^7^	μm^2^
Thickness of the heater	180	μm
Length of the heater	1.12 × 10^4^	μm
Width of the heater	150	μm
Length of the isolation beam	1300	μm
Width of the isolation beam	50	μm
Area of the die attach area	1.26 × 10^5^	μm^2^
Resistivity of the heater	0.005~0.025	Ω·cm

**Table 2:** Residual gas analysis results of the vacuum package.

Gas	H_2_	CO	N_2_	CH_4_	H_2_O	O_2_	C_n_H_m_	Ar	CO_2_	Noble Gas	Total
Pressure (mTorr)	-	-	-	2.2 × 10^−1^	-	-	9.0 × 10^−3^	5.9	-	1.4 × 10^−2^	6.14
Concentration (%)	-	-	-	3.58	-	-	0.15	96.04	-	0.23	100

## Data Availability

The data presented in this study are available from the corresponding author, [K.H.], upon reasonable request.
